# Mesoporous Silica Nanoparticles Improve Oral Delivery of Antitubercular Bicyclic Nitroimidazoles

**DOI:** 10.1021/acsbiomaterials.1c00807

**Published:** 2021-08-31

**Authors:** Chee Wei Ang, Lendl Tan, Zhi Qu, Nicholas P. West, Matthew A. Cooper, Amirali Popat, Mark A.T. Blaskovich

**Affiliations:** † Centre for Superbug Solutions, Institute for Molecular Bioscience, The University of Queensland, St Lucia, Queensland 4072, Australia; ‡ School of Science, Monash University Malaysia, Subang Jaya 47500, Selangor, Malaysia; § School of Chemistry and Molecular Bioscience, The University of Queensland, St Lucia, Queensland 4072, Australia; ∥ Australian Infectious Diseases Research Centre, St Lucia, Queensland 4067, Australia; ⊥ School of Pharmacy, The University of Queensland, Woolloongabba, Queensland 4102, Australia; # Mater Research Institute and Translational Research Institute, The University of Queensland, Woolloongabba, Queensland 4102, Australia

**Keywords:** mesoporous silica nanoparticles, nitroimidazole, solubility, tuberculosis, oral delivery

## Abstract

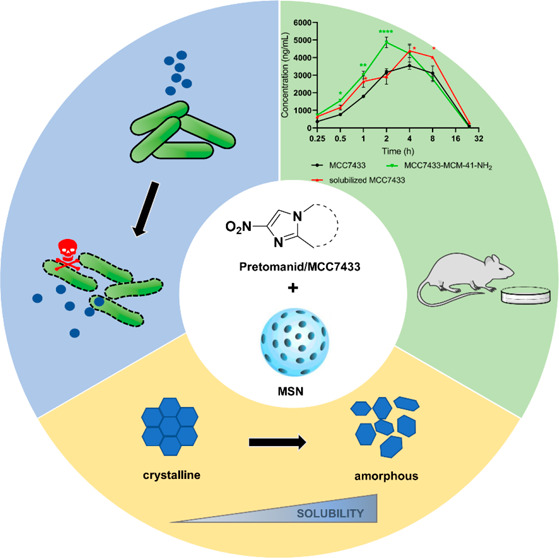

Pretomanid and MCC7433, a novel nitroimidazopyrazinone analog, are promising antitubercular agents that belong to the bicyclic nitroimidazole family. Despite possessing high cell permeability, they suffer from poor aqueous solubility and require specialized formulations in order to be orally bioavailable. To address this limitation, we investigated the use of mesoporous silica nanoparticles (MCM-41) as drug carriers. MCM-41 nanoparticles were synthesized using a sol–gel method, and their surface was further modified with amine and phosphonate groups. A simple rotary evaporation method was used to incorporate the compounds of interest into the nanoparticles, leading to a high encapsulation efficiency of ≥86% with ∼10% loading (w/w). An overall significant improvement of solubility was also observed, and the pharmacological activity of pretomanid and MCC7433 was fully retained when tested in vitro against *Mycobacterium tuberculosis* using these nanocarriers. Amino-functionalized MCM-41 nanoparticles were found to enhance the systemic exposure of MCC7433 in mice (1.3-fold higher *C*_max_) compared to MCC7433 alone. The current work highlights the potential of using nanoparticles such as mesoporous silica as a carrier for oral delivery of poorly soluble antibacterial agents against tuberculosis.

## 1 Introduction

Tuberculosis (TB) caused by the bacillus *Mycobacterium tuberculosis* is one of the world’s deadliest infectious diseases. According to the World Health Organization (WHO), approximately 10 million people suffer from TB every year, with more than 95% of cases and deaths occur in developing countries.^1^ Current therapy against drug-susceptible TB is considered to be effective, but the treatment duration is lengthy and complicated, which leads to poor patient compliance and the emergence of resistant strains.^2^ Drug regimens to treat multidrug-resistant and extensively drug-resistant TB are far from ideal, and many of them are associated with increased toxicity, high cost, and unfavorable side effects.^2,3^ To better eradicate this disease, treatment options that can reduce dosing frequency, shorten treatment duration, and overcome the bacterial resistance are highly desirable.^4^

Solubility plays a significant role in determining the success or failure of a drug candidate once it moves beyond in vitro assays. It is known that compounds with low solubility suffer from poor oral absorption and bioavailability.^5^ Despite the importance of solubility in drug discovery and development, about 40% of the drugs in the market are practically water insoluble,^6^ and many drugs in early-stage development are referred to as “brick-dust”. Poorly soluble drugs tend to have suboptimal systemic exposure after oral administration and therefore require multiple and high dosing to achieve the desired therapeutic concentration. The increased pill burden leads to reduced patient adherence, a key concern when treating infections. This is especially prominent in the area of TB, where both recently approved drugs, delamanid and bedaquiline, are highly lipophilic and have limited solubility. Their high lipophilicity appears to be vital for better penetration into the waxy, lipid-rich mycobacterial cell wall.^7^ It is recommended that these drugs are to be administered with food to increase their oral bioavailability. However, this poses a challenge, as most TB patients are from low-resource settings and many of them are undernourished or suffer from other illnesses that can complicate the drug absorption.^8^

Nanoencapsulation is a promising strategy in drug development especially for potent drugs with suboptimal properties such as poor solubility, low bioavailability, and lack of optimal dosing.^8−10^ However, this approach has been explored sparingly in TB drug research compared to other disease areas such as cancer and other inflammatory diseases. In drug delivery, nanoparticles are generally referred to as colloidal particles with sizes of 10–1000 nm.^11^ This slightly differs to the size defined by the U.S. National Nanotech Initiative, approximately 1–100 nm.^12^ The use of nanoparticles for drug delivery provides several unique advantages.^11,13^ They can deliver drugs to specific targets and achieve controlled release, and because of the nanoscaled size, they can be transported more freely in the body.^13,14^ Importantly, they can improve the solubility and stability of entrapped therapeutic agents, countering some of the liabilities of less optimized candidates.^15−17^ The size of the drug particles can also be reduced to nano- or subnanoscales with the use of nanotechnology. As the drug particle’s size gets smaller, their surface area per volume ratio is increased and thereby the dissolution rate can be enhanced.^18^

Mesoporous silica nanoparticles (MSNs) have recently gained considerable interest as a promising drug carrier. They have uniform and tunable pore size, large surface area and pore volume for high loading capacity, and their surface can be functionalized to accommodate different type of drugs.^19−21^ MSNs have demonstrated high biocompatibility in vivo and appear to be nontoxic in many biological assays.^22−24^ Numerous studies have demonstrated the ability of MSN to enhance drug solubility and dissolution, in particular for Biopharmaceutics Classification System (BCS) class II and IV drugs including antibiotics.^25−27^ Drugs belonging to the BCS class II have poor solubility and high permeability, whereas BCS class IV drugs have poor solubility and poor permeability. MSNs can also act as a selective delivery system for antitubercular drugs, as they have been shown to internalize efficiently in macrophages, the primary host cells of *M. tuberculosis.*(28,29)

The first MSNs were discovered by Mobil researchers in 1992 and were designated as Mobil Crystalline Materials (MCM-41). MCM-41 is one of the most widely explored MSNs for drug delivery. The first study of using MCM-41 as a drug carrier was by using ibuprofen as a candidate. Two different pore sizes of MCM-41 were synthesized, with the larger pore size displaying a higher release rate of ibuprofen.^30^ The hexagonally ordered MCM-41 was also found to improve the drug release of nimodipine, a BCS class II drug.^31^ The use of MCM-41 in BCS class IV drugs was equally effective. Compared to the free drug, the solubility of encapsulated vorinostat within the pristine (unfunctionalized) and functionalized MCM-41 was improved by 2.6–4.3-fold.^17^ Surface functionalization was also shown to affect the loading capacity. The drug loading of alendronate within amine-functionalized MCM-41 was almost three times higher as compared to the pristine material.^32^ All these examples have demonstrated the possibility of modulating the properties of MCM-41 to accommodate different applications.

**Figure 1:**
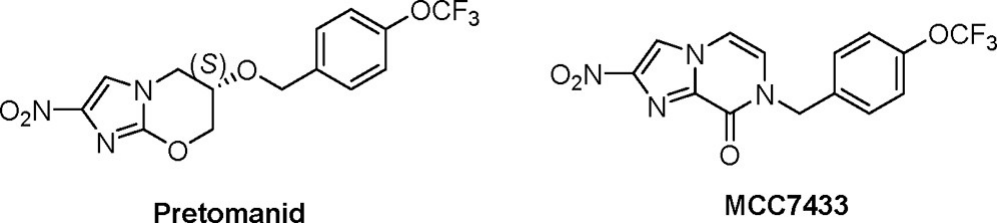
Chemical structures of bicyclic nitroimidazoles, pretomanid, and MCC7433.

In this study, we investigate the use of MCM-41 nanoparticles as drug carriers to improve the physicochemical properties of two poorly soluble antitubercular agents, pretomanid and MCC7433 (Figure 1). Pretomanid is a nitroimidazooxazine that was approved by the U.S. Food and Drug Administration (FDA) in August 2019 as part of a three-drug regimen against drug-resistant tuberculosis.^33^ MCC7433, on the other hand, is a novel nitroimidazopyrazinone that was previously developed within our group, with potent activity against *M. tuberculosis* under both normoxic and hypoxic conditions.^34,35^ This compound also displayed good ADME properties and low cytotoxicity against mammalian cell lines, making it an ideal candidate to be further developed. Similar to other BCS class II drugs, both pretomanid and MCC7433 have low solubility and high permeability.^34^ Here, we describe a simple and efficient rotary evaporation method to encapsulate these compounds into MCM-41 nanoparticles, with high loading capacity and efficiency achieved. These nanoformulations were further characterized for their physical properties, biological activity against *M. tuberculosis* and in vivo pharmacokinetic profile. To the best of our knowledge, this is the first study to prepare nanoformulations of these compounds and test them in vivo after oral delivery.

## 2 Materials and Methods

All the chemicals and reagents were purchased from commercial sources and used without further purification. Pretomanid was purchased from Toronto Research Chemicals Inc. (Ontaria, Canada). MCC7433 was previously reported and was synthesized via a five-step reaction.^34^ Standard drug isoniazid was purchased from Sigma-Aldrich (Missouri, United States).

### 2.1 Synthesis of MCM-41

MCM-41 was synthesized according to a previously reported method.^17^ Briefly, 1 g of cetyltrimethylammonium bromide (CTAB) was dissolved in 480 mL of deionized water, followed by addition of 3.5 mL of 2 M NaOH. The reaction mixture was heated up to 80 °C and 6.7 mL of tetraethyl orthosilicate (TEOS) was added slowly. The reaction was continued for 2 h at 80 °C with constant stirring. The resulting product was filtered, washed with deionized water, and dried overnight at 60 °C in the oven. The surfactant templates were removed by calcination at 550 °C for 5 h in a muffle furnace, at a temperature ramp rate of 5 °C/min for the up-ramp and 10 °C/min for the down-ramp.

### 2.2 Surface Functionalization of MCM-41

The surface of MCM-41 was modified by introducing amine and phosphonate groups, respectively.^17^ For amine functionalization, 400 mg of MCM-41 particles was dispersed in 60 mL of toluene and stirred at 50 °C for 30 min. To this suspension was added 400 μL of (3-aminopropyl)triethoxysilane (APTES) and the solution was refluxed overnight at 110 °C. The resulting particles were then centrifuged at 15 000 rpm for 10 min and washed with ethanol three times before drying overnight at 60 °C. For phosphonate functionalization, 400 mg of MCM-41 particles were dispersed in 65 mL of deionized water containing 400 μL of 3-(trihydroxysilyl)propyl methylphosphonate (THMP). The pH of the THMP solution was adjusted to pH 5–6 before addition of MCM-41 and was refluxed overnight at 100 °C. The functionalized particles were collected by centrifugation at 15 000 rpm for 10 min and washed with deionized water and ethanol three times. The material was then dried overnight at room temperature for further analysis.

### 2.3 Encapsulation of Bicyclic Nitroimidazoles into MSNs

Loading of pretomanid and MCC7433 was performed using a rotary evaporation method as described previously.^36^ To achieve a theoretical loading of 10%, 10 mg of pretomanid was first dissolved in 10 mL of ethanol. Ninety milligrams of MCM-41 (pristine or functionalized) in 10 mL of ethanol was then added to the drug solution and dispersed using a bath sonicator for 5 min. The mixture was allowed to stir for 2 h at 37 °C before removing all the solvent slowly by rotary evaporator at 45 °C. The obtained loaded sample was then transferred to a vacuum oven and dried overnight at 45 °C. To encapsulate 10% of MCC7433, we used the same method except the loading was performed in ethyl acetate/methanol (1:1, v/v).

### 2.4 Material Characterization

#### 2.4.1 Differential Scanning Calorimetry (DSC) and Thermogravimetric Analysis (TGA)

Differential scanning calorimetry (DSC) was conducted to characterize the physical state of pretomanid and MCC7433 before and after loading into nanoparticles. Thermogravimetric analysis (TGA) was used to calculate the loading capacity of compounds in MSNs and to determine the percentage of grafted moieties (amino and phosphonate groups) onto the MCM-41 mesopores. Around 5 mg of nanoparticles was accurately weighed on an alumina crucible and analyzed using a Mettler Toledo instrument (TGA/DSC 2, Columbus, OH, USA) under airflow of 20 mL min^–1^ with a heating rate of 10 °C min^–1^, from 50 to 900 °C.

#### 2.4.2 Transmission Electron Microscopy (TEM)

TEM images were taken using a HITACHI HT7700B microscope (Tokyo, Japan) operated at 80 kV. The nanoparticles were dispersed in ethanol (MCM-41), sonicated, and dried as a film on a carbon-coated copper grid.

#### 2.4.3 Nitrogen Sorption

Nitrogen adsorption/desorption isotherms were measured using a Tri-Star II 3020 nitrogen adsorption system (Norcross, GA, USA) to measure the surface area and porosity of particles before and after loading onto MSNs. The samples were weighed around 50–70 mg and degassed overnight on a vacuum line prior to analysis. Surface area was calculated based on Brunauer–Emmett–Teller (BET) method using adsorption data at relative pressure (*p*/*p*_0_),^37^ whereas pore size and volume were derived from the Barrett– Joyner–Halenda (BJH) method.^38^

#### 2.4.4 Dynamic Light Scattering (DLS) and Zeta Potential

The mean particle size, polydispersity index (PDI), and zeta potential were measured using dynamic light scattering at 25 °C with Nano series ZS instrument (Malvern, Nano-ZS, ATA Scientific, Taren Point, Australia). The particles were suspended in water or PBS (pH 7.4) and sonicated for 20 min prior to measurements.

### 2.5 Loading Capacity and Encapsulation Efficiency

The amount of compound loaded into the MSNs was determined using TGA by calculating the weight loss of samples as a function of temperature (200–900 °C) in comparison with the blank MSNs.
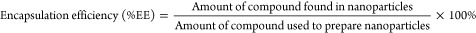

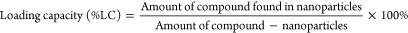


### 2.6 Aqueous Solubility Determination

The solubility of pretomanid and MCC7433 was measured before and after loading into nanoparticles by using Shimadzu LCMS-2020 (Kyoto, Japan) with a variable-wavelength UV–visible detector. Briefly, 0.5 mg of compound equivalent particles (or 5 mg of 10 wt % compound loaded nanoparticles) were dispersed in 750 μL of deionized water. Samples were incubated at 37 °C on a rotator for 24 and 48 h. Samples were then centrifuged at 12 000 rpm for 20 min. The supernatant was collected and centrifuged for another round at 12 000 rpm, 20 min before being analyzed by LC-UV. The mobile phase was 0.05% formic acid in water (solvent A) and 0.05% formic acid in acetonitrile (solvent B). LC-MS condition: column Zorbax Eclipse XDB-Phenyl, 3.0 × 100 mm, 3.5 μm; column temperature: 40 °C; flow: 1 mL/min; injection volume: 5 μL; gradient timetable: 0.00 min, 5% B; 0.50 min, 5% B; 3.00 min, 100% B; 4.2 min, 100% B; 5.00 min, 5% B.

### 2.7 *M. tuberculosis* H37Rv Minimum Inhibition Assays

The minimum inhibitory concentration with >90% inhibition (MIC_90%Inhib_) study was performed using a resazurin reduction microplate assay as previously described.^34,39^ The plates were incubated for 5 days at 37 °C in a humidified incubator prior to the addition of 30 μL of a 0.02% resazurin solution and 12.5 μL of 20% Tween-80 to each well. After 24 h of incubation (37 °C), sample fluorescence was measured on a FLUOstar Omega fluorescent plate reader (BMG LABTECH, Ortenberg, Germany) with an excitation wavelength of 530 nm and emission read at 590 nm. The assays were performed in replicate on independent occasions (*n* = 4–6). To prevent possible interferences due to the nanoparticles, blank nanoparticles (MCM-41, MCM-41-NH_2_, and MCM-41-PO_3_^–^) were used as negative controls. A standard drug, isoniazid, was used as positive control. MIC_90%Inhib_ was assessed as the lowest concentration that resulted in >90% inhibition of *M. tuberculosis* growth.

### 2.8 In Vivo Pharmacokinetic Study

Mouse pharmacokinetic studies were conducted by WuXi AppTec Co., Ltd. (Shanghai, China). The experimental procedures were approved by the Institutional Animal Care and Use Committee of WuXi AppTec Co., Ltd. (Protocol number PK01–001–2019v1.0). MCC7433 and its nanoformulations were dispersed in water by a probe homogenizer at equivalent concentrations of 5 mg/mL. For comparison, a fully solubilized formulation of MCC7433 was prepared in 10% DMSO and 90% PEG400 and was dosed at the same concentration. These samples were then administered to CD-1 male mice (*n* = 3) via oral gavage at 20 mg/kg. Approximately 30 μL of blood was taken via the submandibular or saphenous vein for the first several time points (0.25, 0.5, 1, 2, 4, and 8 h). Blood samples at the last time point (24 h) were collected via cardiac puncture while the mouse was under CO_2_ anesthesia. All blood samples were transferred into prechilled microcentrifuge tubes containing 2 μL of K_2_-EDTA (0.5 M) as anticoagulant and placed on ice until centrifugation. Harvested blood samples were centrifuged at 7000 rpm at 4 °C for 10 min. Plasma was then collected, frozen over dry ice, and stored at −70 °C until LC-MS/MS analysis. To process the samples prior to analysis, we quenched an aliquot of 5 μL with 300 μL of acetonitrile containing internal standards (labetalol, tolbutamide, verapamil, dexamethasone, glyburide, and celecoxib, 100 ng/mL for each). The mixture was vortex-mixed and centrifuged for 15 min at 4000 rpm and 4 °C. Supernatant (50 μL) was transferred to a 96-well plate and centrifuged again for 5 min at 4000 rpm and 4 °C before injecting to LC-MS/MS. Calibration standards was prepared at 1–3000 ng/mL for quantitation. Mobile phase was 0.1% formic acid and 2 mM ammonium formate in water/acetonitrile (v:v, 95:5) (solvent A) and 0.1% formic acid and 2 mM ammonium formate in acetonitrile/water (v:v, 95:5) (solvent B). LC-MS/MS condition: column ACQUITY UPLC HSS T3 1.8 μm 2.1 × 50 mm; column temperature: 60 °C; flow: 0.6 mL/min; gradient timetable: 0.00 min, 10% B; 1.20 min, 90% B; 1.40 min, 90% B; 1.41 min, 10% B; 1.50 min, 10% B. Pharmacokinetic data were calculated using *Phoenix WinNonlin* 6.3 (Certara, Princeton, NJ).

## 3 Results and Discussion

### 3.1 Preparation and Characterization of MCM-41 Nanoparticles

**Figure 2:**
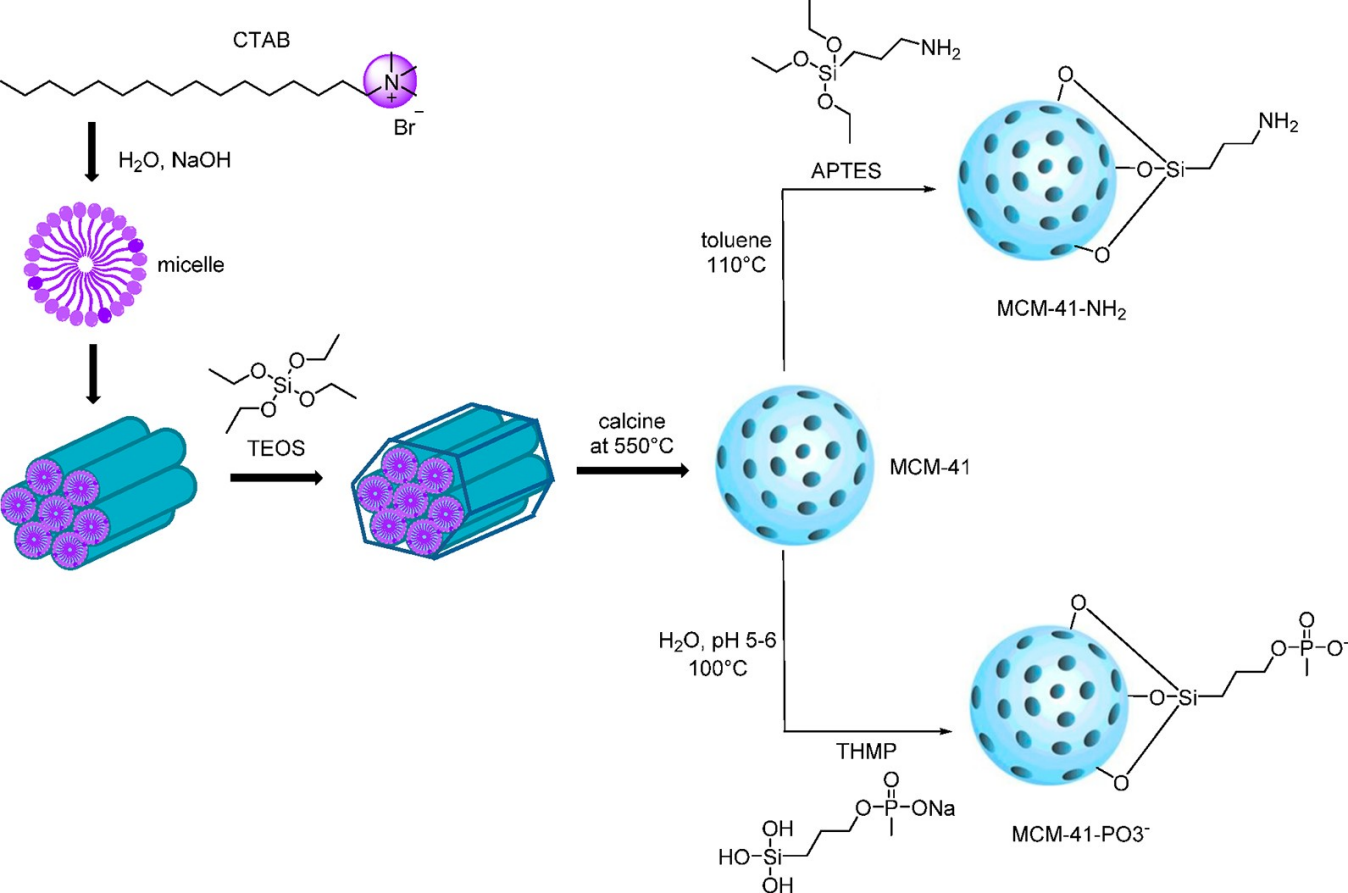
Synthesis of functionalized MCM-41 through postsynthetic grafting method.

The synthesis of MCM-41 was achieved via a sol–gel process, using surfactant cetyltrimethylammonium bromide (CTAB) as the structure-directing agent, tetraethyl orthosilicate (TEOS) as the silica source, and NaOH as base catalyst. This process involved hydrolysis and condensation of the silica precursor to form the mesoporous structure.^40^ Initially, the long-chained surfactant molecules arranged themselves as a micelle, with an inner core consisting of hydrophobic tails. The positively charged micelles then interacted electrostatically with the negatively charged silicate species to form a tubular silica around the micelles.^19^ Calcination was used to remove the surfactant template as the solvent extraction method can be incomplete.^41^ To functionalize MCM-41, we employed a postsynthetic grafting method by using organosilane surface modifiers (i.e., APTES for amine groups and THMP for phosphonate group). Functional groups were introduced to the particles by silylation of free and germinal silanol groups on the surface of MCM-41.^42,43^ This provides a different surface charge that can be used to manipulate the drug release and delivery under different pH conditions. The preparation of both pristine and functionalized MCM-41 is illustrated in a simplified graphic in Figure 2.

**Figure 3:**
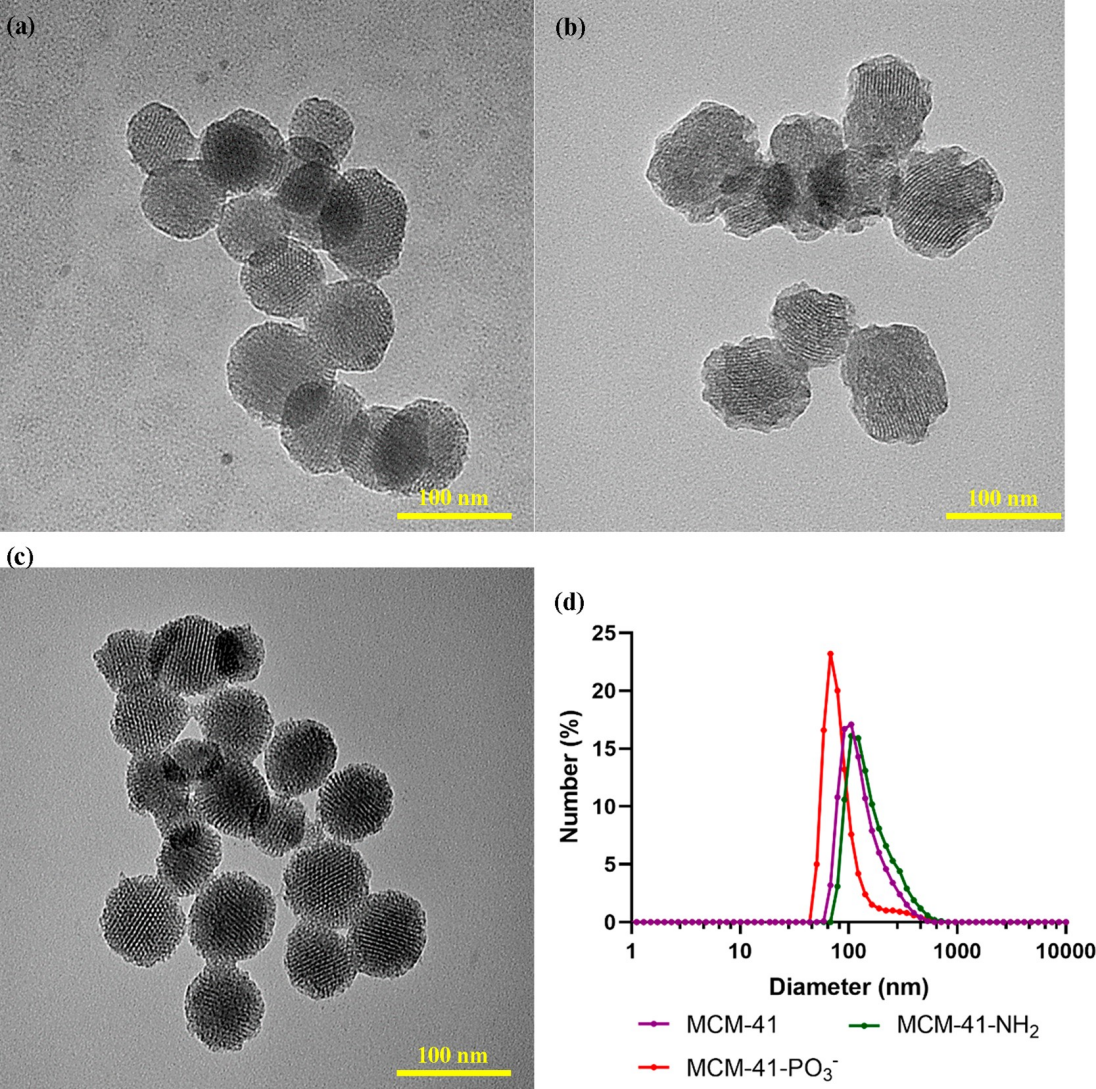
TEM images of (a) MCM-41, (b) MCM-41-NH_2_, and (c) MCM-41-PO_3_^–^; (d) their DLS particle size distribution in aqueous solution.

The morphology and size of the synthesized particles were first determined by TEM. As shown in Figure 3, the MCM-41 particles were mostly spherical with a parallel channel-like, hexagonal pore arrangement comparable to that in a previous report.^44^ This suggests the successful synthesis of MCM-41. Amino- and phosphonate-modified MCM-41 revealed a similar honeycomb structure as the pristine particles, although the outer surface of amino-functionalized MCM-41 (MCM-41-NH_2_) was slightly irregular. This disordered structure might be attributed to the presence of amino groups on the surface. From zeta potential measurement, MCM-41 showed a negative surface potential of −20 mV due to the presence of silanol groups. Functionalization of these particles shifted their zeta potential, giving a positive zeta of +32 mV for MCM-41-NH_2_ and a more negative value of −46 mV for phosphonate functionalized MCM-41 (MCM-41-PO_3_^–^), confirming the successful grafting of the nanoparticles. From the DLS study in water, the number mean size of ∼100–200 nm was observed for all the particles. The size of particles measured by DLS was slightly larger compared to TEM, which could be due to the presence of a hydration layer surrounding the particles and possible aggregation in solution.^45,46^ Pristine MCM-41 showed a narrow size distribution, which can be reflected from its low polydispersity index (PDI = 0.148). After functionalization, the PDI was increased to ∼0.3–0.5. (Table 1).

**Table 1:** Particle Size, PDI, and Zeta Potential of Pristine and Functionalized MCM-41

sample	*Z* average size (*d*, nm)	PDI	number mean (nm)	zeta potential (mV)
MCM-41	188 ± 2.12	0.15 ± 0.04	146 ± 8.15	–19.7 ± 1.08
MCM-41-NH_2_	252 ± 10.1	0.31 ± 0.03	174 ± 16.7	+31.5 ± 0.65
MCM-41-PO_3_^–^	276 ± 14.6	0.47 ± 0.04	96.2 ± 10.6	–46.4 ± 2.95

**Figure 4:**
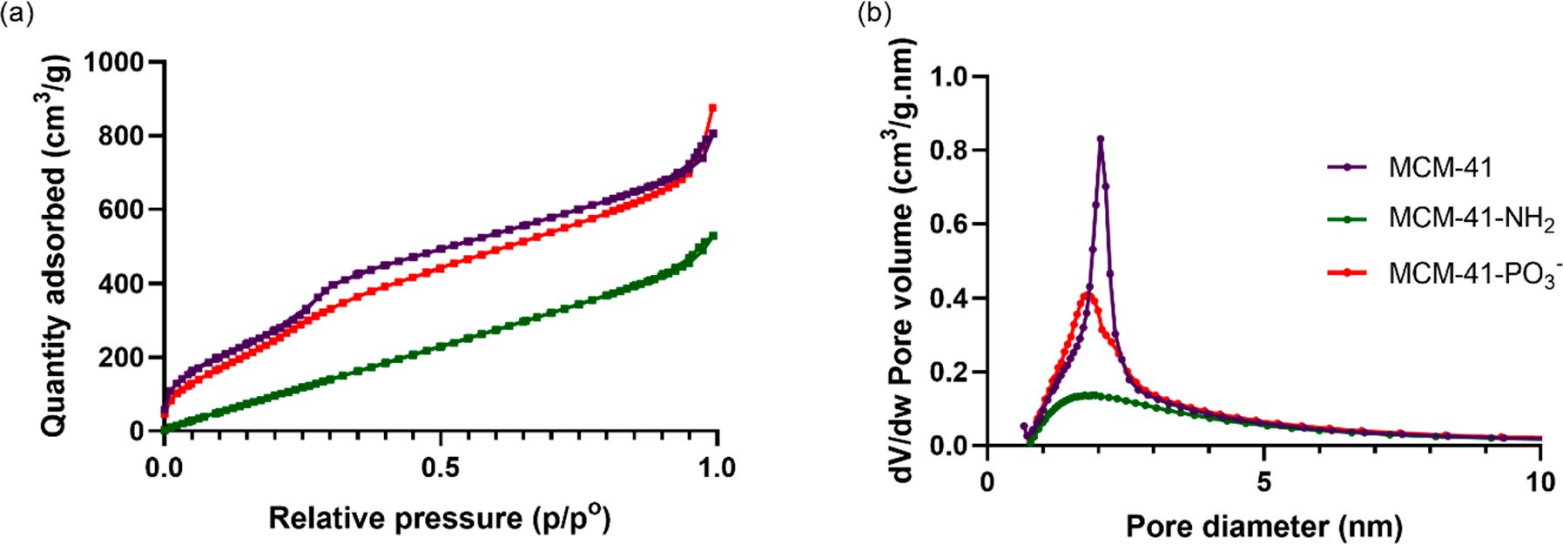
(a) N_2_ sorption isotherms and (b) BJH pore size distribution of MCM-41, MCM-41-NH_2_, and MCM-41-PO_3_^–^.

**Figure 5:**
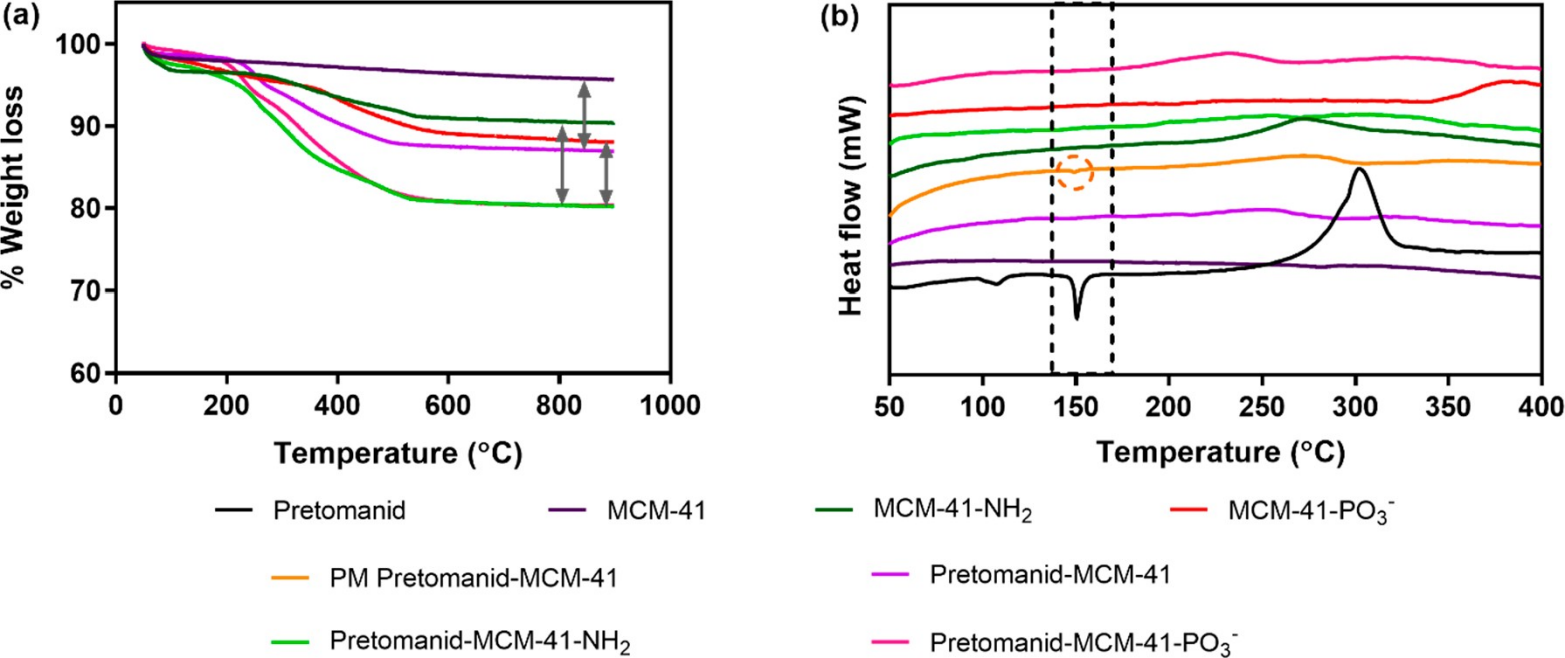
Graphs of (a) TGA and (b) DSC of 10% loading of pretomanid in pristine and functionalized MCM-41. PM pretomanid-MCM-41 refers to the physical mixing of pretomanid and MCM-41 at the same loading ratio, with a small peak observed near the melting point of pretomanid.

Nitrogen (N_2_) sorption analysis was performed to assess the pore features of pristine and functionalized MCM-41. As depicted in Figure 4, pristine MCM-41 showed a typical type IV isotherm and a distinct step at relative pressure (*p*/*p*_0_) of ∼0.2–0.4, which is a characteristic pattern of MCM-41 type mesoporous materials.^17,47^ Although the shape of isotherms of MCM-41-NH_2_ and MCM-41-PO_3_^–^ changed slightly due to the partially filling of pores during functionalization, they were still maintained as type IV. This indicated that the ordered structure of MCM-41 remained, and the pores were not blocked completely. Upon functionalization, there was a reduction in BET surface area and total pore volume as presented in Table 2. The surface area and pore volume of MCM-41-NH_2_ decreased by ∼50% compared to unfunctionalized MCM-41, whereas MCM-41-PO_3_^–^ showed only ∼5% reduction. This is expected, as functionalization by the postsynthetic grafting method can happen both inside or outside of the pores.^48^ The BJH pore size of the pristine MCM-41 (2.04 nm) was also reduced to 1.86–1.89 nm by functionalization. From the TGA analysis, the functionalized MCM-41 showed higher weight loss compared to the pristine MCM-41 (Figure 5a). The weight loss near 100 °C was attributed to the loss of adsorbed moisture, whereas the weight loss above 200 °C corresponded to the thermal decomposition of functional groups attached to the pore walls.^49^ On the basis of the weight loss between 200 and 900 °C, the percentage of grafting of amino and phosphonate groups was determined to be 4.5 and 6.4% of the total mass.

**Table 2:** Physical Properties of Pristine and Functionalized MCM-41 Obtained by BET

sample	specific surface area, *S*_BET_ (m^2^/g)	total pore volume, *V*_p_ (cm^3^/g)	pore size (nm)
MCM-41	503.40	1.053	2.04
MCM-41-NH_2_	251.84	0.539	1.89
MCM-41-PO_3_^–^	477.34	1.011	1.86

### 3.2 Drug Loading on MCM-41

**Figure 6:**
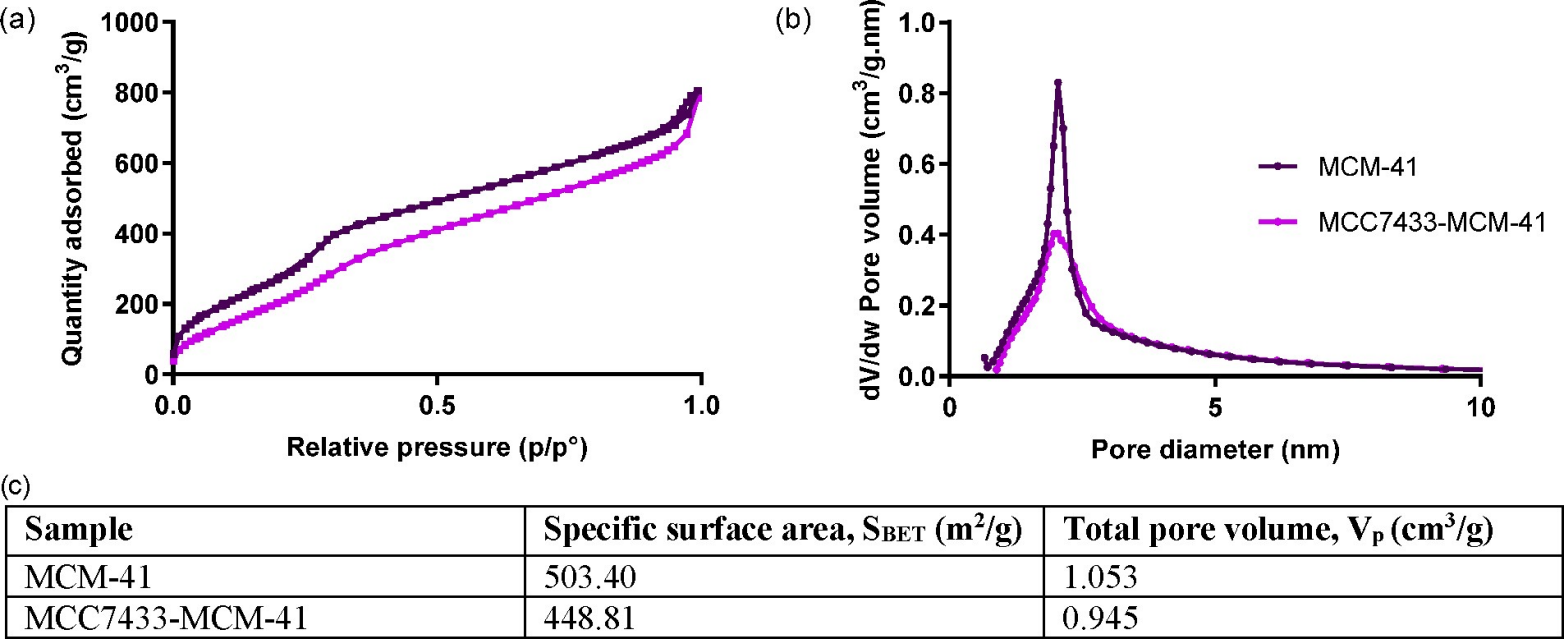
(a) N_2_ sorption isotherms, (b) BJH pore size distribution, and (c) physical properties of MCM-41 before and after loading with MCC7433.

A rotary evaporation method was selected to load the compounds of interest into mesoporous silica due to the high loading efficiency of this procedure.^50^ The solvent (ethanol or ethyl acetate/methanol) was chosen based on the solubility of the drug and to facilitate the drug-pore interactions over drug-solvent interactions.^51^ All the particles were loaded with pretomanid and MCC7433 at a theoretical loading of 10% w/w. The mixture of drugs and particles were dispersed uniformly under ultrasonication before stirring for 2 h at 37 °C to achieve adsorption equilibrium. Solvent was slowly removed by evaporation, causing a concentration difference between the pores and the outer solution that enabled the drugs to be diffused into the mesoporous material. This process also transforms the adsorbed crystalline molecules to an amorphous state. Because of its constrained environment and lower Gibbs free energy, it can suppress the recrystallization of the amorphized drugs.^52^ DSC analysis is a useful tool for characterizing crystallinity and thermal stability. From the DSC curve of pure pretomanid in Figure 5b, a single sharp endothermic peak was seen at 150 °C that indicates the melting point of its crystalline form, with a larger exothermic peak at ∼305 °C due to thermal decomposition of pretomanid. When pretomanid was physically mixed with MCM-41 at the same loading ratio (PM pretomanid-MCM-41 in Figure 5b), a small peak near the melting point temperature was still present, showing the crystalline melting point. However, after loading into the pore channels of MSNs, this characteristic peak disappeared, indicating that the drug was in an amorphous state, losing its crystallinity. A similar observation was found when MCC7433 was loaded into the particles, in which its crystalline endothermic melting peak at 267 °C disappeared, confirming confinement of MCC7433 within nanopores of MCM-41 in an amorphous form.^21,53^ To further determine whether the drug molecules were encapsulated within the mesopores or just adsorbed on the outer surface, we performed N_2_ sorption analysis to characterize their pore features after loading. As shown in Figure 6, the specific surface area and pore volume of MCM-41 were decreased upon loading of MCC7433. This indicates that the molecules were partially filling the pores and successfully confined within the channel.

**Figure 7:**
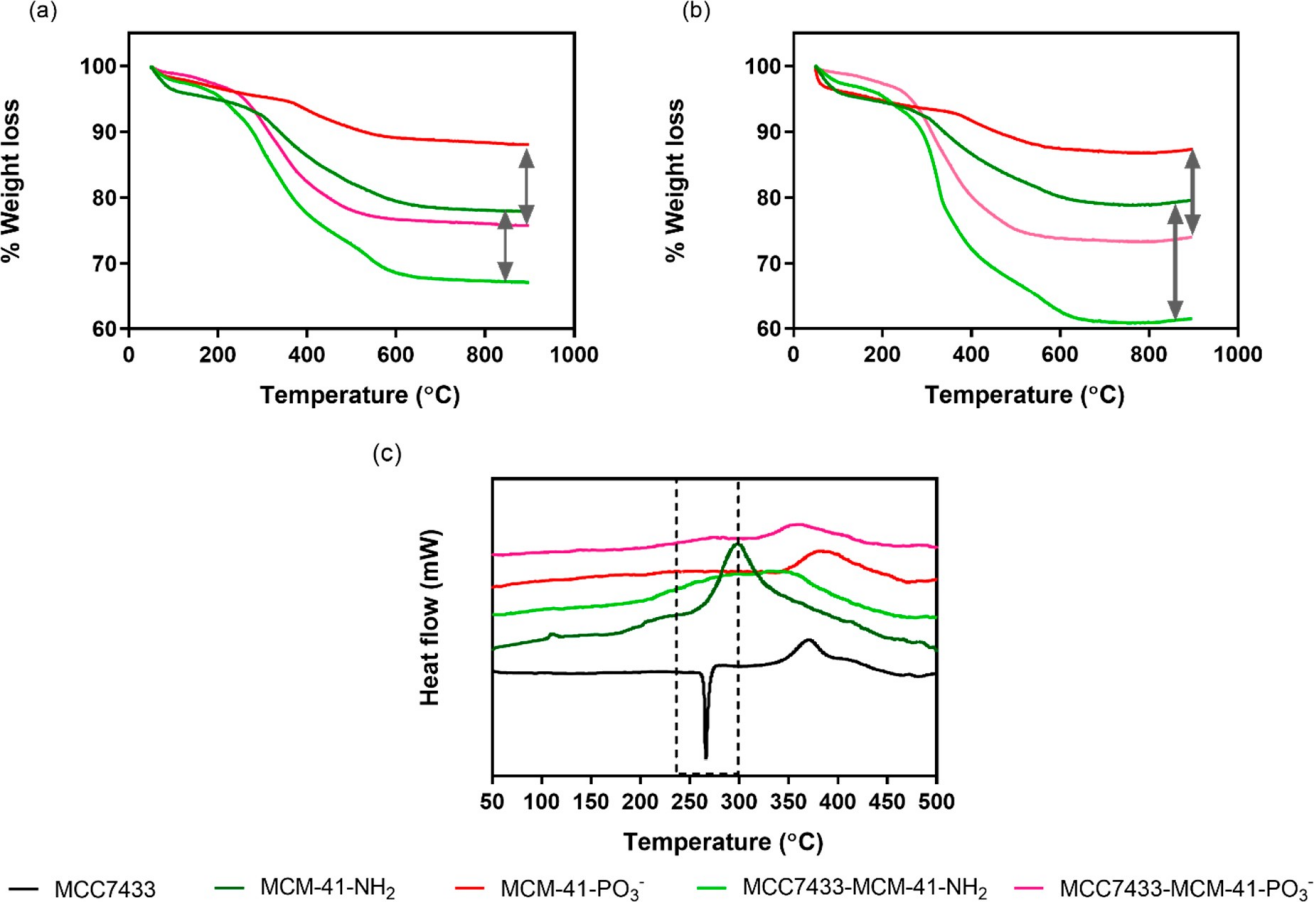
Graphs of TGA and DSC of MCC7433 loaded in functionalized MCM-41. The loading amount was determined from TGA with (a) 10% w/w and (b) 20% w/w of MCC7433. (c) DSC profile of free and loaded MCC7433.

The concentration of compounds loaded into the MSNs was determined using TGA by calculating the weight loss between 200 and 900 °C. Overall good loading efficiency was achieved for both pristine and functionalized MCM-41. With a theoretical loading of 10% w/w, the loading capacity of pretomanid as determined by TGA was 9.0, 9.4, and 8.6% in MCM-41, MCM-41-NH_2_, and MCM-41-PO_3_^–^ (Figure 5a). The loading capacity of MCC7433 at the same amount was slightly higher, with 10–13% of MCC7433 loaded into the functionalized particles. A theoretical loading of 20% w/w was also attempted on MCC7433, with TGA showing 19 and 16% of compound loaded into MCM-41-NH_2_ and MCM-41-PO_3_^–^ (Figure 7). This has demonstrated the high loading capacity of MCM-41. In most cases, there was no significant change in particle size and zeta potential after loading (Table 3). However, the PDI for loaded MCM-41 increased from 0.148 to 0.291–0.420 which could be due to release of the drug during measurement or an increase in surface hydrophobicity of the overall particles due to drug loading. This observation is also consistent with the previous study done by our group.^21,54^ From representative TEM images of pretomanid-MCM-41 and MCC7433-MCM-41 (Figure S1), we demonstrated that the loading procedures did not impact on the mesoporous structure of the nanoparticles.

**Table 3:** Particle Size, PDI, and Zeta Potential of Pretomanid and MCC7433-Loaded MCM-41

sample	*Z* average size (*d*, nm)	PDI	number mean (nm)	zeta potential (mV)
pretomanid-MCM-41	250 ± 2.68	0.42 ± 0.07	120 ± 2.52	–24.1 ± 0.252
pretomanid-MCM-41-NH_2_	268 ± 9.66	0.29 ± 0.03	154 ± 44.0	+33.3 ± 1.00
pretomanid-MCM-41-PO_3_^–^	208 ± 4.65	0.31 ± 0.02	129 ± 5.51	–36.1 ± 2.27
MCC7433-MCM-41	185 ± 3.29	0.29 ± 0.03	104 ± 9.79	–28.0 ± 3.61
MCC7433-MCM-41-NH_2_	258 ± 13.7	0.34 ± 0.07	162 ± 8.75	+32.4 ± 1.59
MCC7433-MCM-41-PO_3_^–^	176 ± 2.60	0.22 ± 0.03	109 ± 8.21	–38.0 ± 0.635

### 3.3 Aqueous Solubility

**Figure 8:**
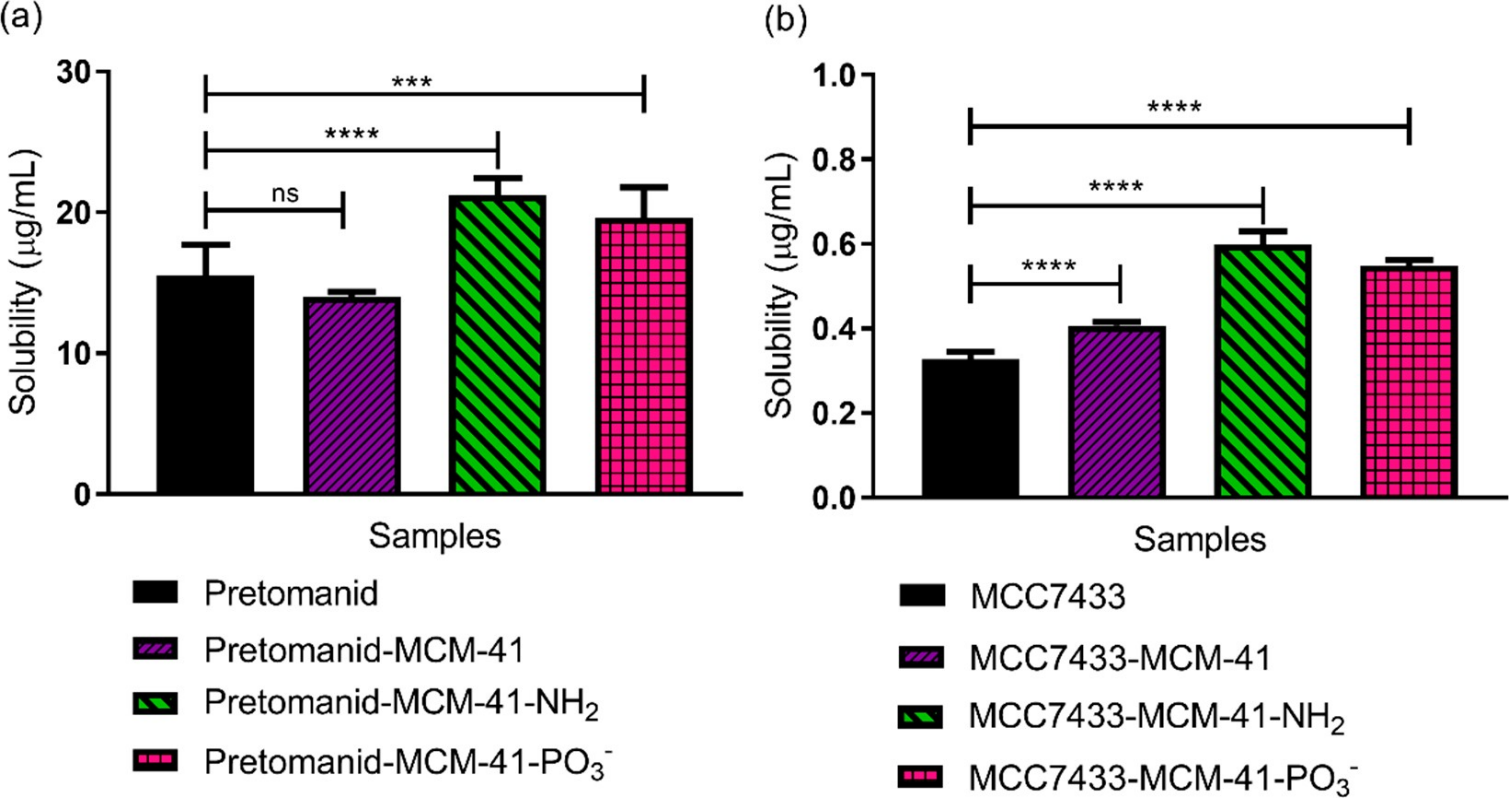
Saturated solubility of free and encapsulated (10% w/w) (a) pretomanid and (b) MCC7433 in water (*n* = 3 ± SD). Solubility of pretomanid-MCM-41-NH_2_ and MCC7433- MCM-41-NH_2_ was 21.2 and 0.60 μg/mL, which was 1.4- and 1.8-fold higher than their free form. *P* value was determined by one-way ANOVA, followed by a posthoc Dunnett’s test: *P* > 0.05, not significant, ns, *** *P* < 0.001, **** *P* < 0.0001.

Oral administration is one of the most convenient and preferred routes of drug delivery because of its cost effectiveness, high patient compliance, and ease of administration. It is particularly important for delivery of medications in low- and middle-income countries, where access to intravenous or subcutaneous injections is much more difficult. However, a major hurdle for oral drug design lies with overcoming poor bioavailability and absorption. Drugs with poor aqueous solubility and low dissolution rates in gastrointestinal fluids often lead to insufficient bioavailability, especially for BCS class II drugs.^5,55^ Therefore, one of our goals was to improve the solubility of pretomanid and MCC7433 through nanoparticle formulation. After 24 h of incubation at 37 °C, the saturated water solubility of free pretomanid and MCC7433 was determined as 15.5 and 0.33 μg/mL, respectively. When these nitroimidazoles were encapsulated in nanoparticles (10% w/w), their solubility increased for most examples (Figure 8). This could be explained from the amorphous character of the encapsulated drugs compared to their crystalline state (as seen in the DSC thermograms), reducing the thermodynamic barrier to dissolution.^56^ Amino-functionalized MCM-41 surpassed other MSNs in enhancing the solubility of pretomanid and MCC7433, giving 1.4- and 1.8-fold improvements over nonencapsulated compounds. To ensure that equilibrium has been achieved, we also measured the concentration of free and loaded pretomanid in solution at 48 h, with no significant difference in solubility was observed at both 24 and 48 h (Figure S3).

### 3.4 Antimycobacterial Activity

To investigate whether the loading and release of compounds from nanoparticles could affect their antimycobacterial activity, we determined the inhibitory effect of free and encapsulated pretomanid and MCC7433 against the *M. tuberculosis* H37Rv strain using a resazurin-based microtiter assay. This study was focused on MCM-41-NH_2_, as this nanocarrier showed the best potential for improving the aqueous solubility of the tested bicyclic nitroimidazoles. As shown in Table 4, the encapsulated pretomanid and MCC7433 that were dispersed in assay media were identified to have comparable inhibitory activity with their free forms that were predissolved in DMSO, with MIC_90_ = 0.25 μg/mL. This confirms that the compounds still maintained their pharmacological activity when delivered through the carriers and were effectively released into solution. Blank MCM-41-NH_2_ nanoparticles were found to be inactive up to concentrations as high as 2880 μg/mL, confirming that the observed bioactivity of the nanoformulation was from the compounds itself.

**Table 4:** Minimum Inhibitory Concentration with >90% Inhibition (MIC_90%Inhib_) of Free and Nanoformulated Pretomanid and MCC7433 against *M. tuberculosis* H37Rv Strain (*n* = 4–6)

samples	MIC_90%Inhib_ H37Rv (μg/mL)
MCM-41-NH_2_	>2880
pretomanid (predissolved in DMSO)	0.25
pretomanid-MCM-41-NH_2_	0.25
MCC7433 (predissolved in DMSO)	0.25–0.5
MCC7433-MCM-41-NH_2_	0.25
isoniazid	0.04

### 3.5 Pharmacokinetic (PK) Profile

As oral dosing is the preferred administration route for TB drugs, the PK properties of MCC7433 and its nanoformulation were determined following oral gavage in mice at a single nominal dose of approximately 20 mg/kg. Previous studies done by others have suggested the potential of MSNs in enhancing the maximal serum concentration (*C*_max_) and bioavailability of poorly soluble drugs.^57^ In this study, the amino-functionalized MCM-41 was investigated, as it provided the highest improvement of solubility compared to other nanoformulations. MCC7433-MCM-41-NH_2_ was dosed as a homogeneous suspension in water, whereas the native MCC7433 was dosed as a suspension in aqueous solution, though in this case it was not completely dispersed, with some remaining adhered to the vial. A fully solubilized formulation of MCC7433 in 10% DMSO and 90% PEG400 was also prepared and dosed at the same concentration as a positive control.

**Figure 9:**
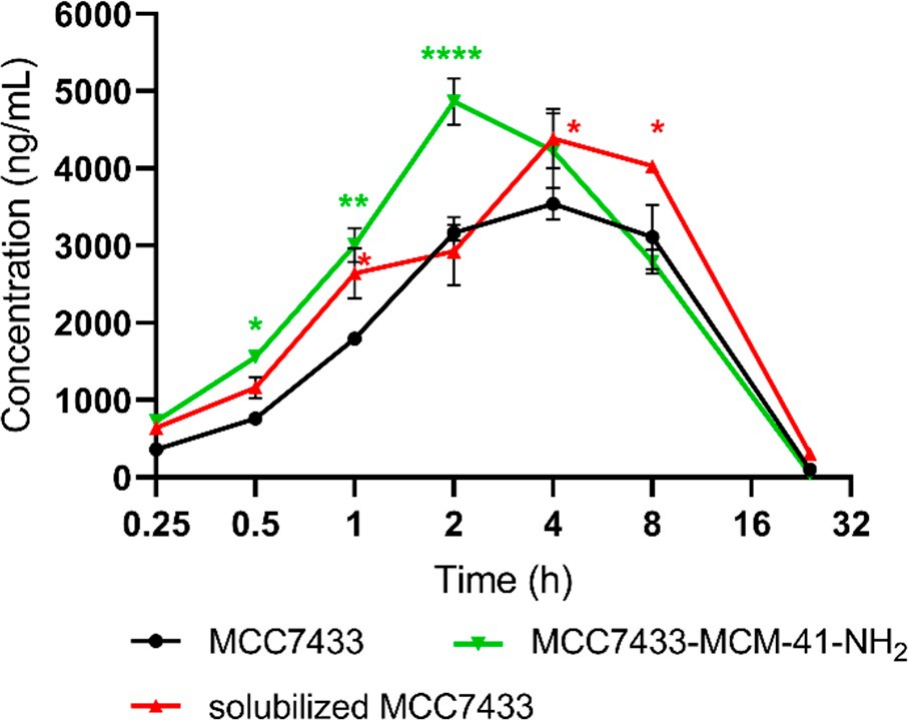
Mean plasma concentration–time curve of free MCC7433 (black, homogeneous aqueous suspension), MCC7433-MCM-41-NH_2_ 10% w/w (green, nonhomogeneous aqueous suspension), and solubilized MCC7433 (red, formulated in 10% DMSO/90% PEG400) after 20 mg/kg of oral administration. This curve represents the normalized apparent plasma concentrations calculated by multiplying the measured levels by the ratio of nominal dose/administered dose (*n* = 3, mean ± SEM). *P* value was determined by two-way ANOVA, followed by posthoc Dunnett’s test: *P* > 0.05, not significant, ns, * *P* < 0.05, ** *P* < 0.01, *** *P* < 0.001, **** *P* < 0.0001.

On the basis of the nominal concentration dosed, the nanoparticle formulation of MCC7433 resulted in an apparent enhancement of the PK profile compared to the nonformulated aqueous solution of MCC7433. It should be noted that the native MCC7433 was not completely dispersed and the actual administered dose (as determined by analytical measurement of the administered solution) was not equivalent between the tested samples (Table 5). Therefore, we reanalyzed the PK data by normalizing the peak plasma concentrations based on the actual dosing, by multiplying the measured plasma concentrations by the ratio of nominal dose/actual administered dose (Figure 9) (e.g., if the administered dose was half of what was expected, this adjustment would give an apparent plasma concentration double the measured plasma concentration). Although this adjustment assumes that plasma levels are linearly proportional to administered dose, it allows for an estimate of the comparative oral availability of the different delivery systems, based on the delivered dose.

**Table 5:** Pharmacokinetic Profile of Unformulated MCC7433, MCC7433-MCM-41-NH_2_, and Solubilized MCC7433 in DMSO/PEG400 Following Oral Administration (PO)^a^

PK parameters	MCC7433	MCC7433-MCM-41-NH_2_ (10% w/w)	solubilized MCC7433 in DMSO/PEG400
nominal dose (mg/kg)	20	200 (equivalent to 20 mg/kg of MCC7433)	20
administered dose (mg/kg)	7.10	64 (equivalent 6.4 mg/kg of MCC7433)	15.2
*C*_max_ (μg/mL)	3.77	4.88	4.47
AUC_0-last_ (μg.h/mL)	37.2	39.1	51.2
*T*_max_ (h)	5.33	2.67	5.33
*T*_1/2_ (h)	3.21	2.98	4.78

a Data are normalized to a dose equivalent to 20 mg/kg of MCC7433 based on the actual dosing, as determined by analytical measurement of the administered solution. AUC_0-last_, area under the concentration–time curve from initial to the last time point; *C*_max_, maximum plasma concentration; *T*_1/2_, elimination half-life; *T*_max_, time to reach *C*_max_.

After normalization, it was noted that mesoporous silica nanoparticles enhanced the PK properties of MCC7433. From Figure 9, the mean plasma concentrations of MCC7433-MCM-41-NH_2_ at various time points were significantly higher than the nonformulated MCC7433. This carrier also significantly improved the *C*_max_ of MCC7433 by 1.3-fold, reaching a peak plasma concentration of 4.88 μg/mL. This value was comparable to the levels reached by the solubilized form in DMSO/PEG400 (*C*_max_ = 4.47 μg/mL), a formulation that would not be acceptable for human use. The area under curve from the initial to the last time point (AUC_0-last_) was slightly enhanced from 37.2 to 39.1 μg.h/mL. MCC7433-MCM-41-NH_2_ gave an overall exposure, as measured by AUC_0-last_, that was better than aqueous MCC7433, albeit not quite as good as the DMSO-solubilized form. This demonstrated that the MSNs can facilitate the dissolution of MCC7433 in the gastrointestinal fluid, creating a higher concentration gradient between the gastrointestinal lumen and blood.^57^ Although some studies showed that amino-functionalized MSNs have potential in delaying the drug release.^58^ this nanoformulation showed a reduced *T*_max_ of 2.7 h compared to 5.3 h for solubilized MCC7433, suggesting that MCM-41-NH_2_ could improve the absorption of MCC7433 after oral administration. The increase in gastrointestinal absorption could also lead to enhanced bioavailability. At 24 h, the plasma concentration returned close to baseline.

## 4 Conclusions

This study describes investigations into the use of MCM-41 type silica nanoparticles as a carrier for poorly soluble heterocyclic nitroimidazole compounds targeting tuberculosis. MCM-41 nanoparticles were prepared using a sol–gel protocol, forming a highly ordered mesoporous structure with a targeted nanometer size range. Modification of the properties of these nanoparticles via functionalization of the particle surface was achieved through a postsynthetic grafting method, in which either terminal amino or phosphonate groups were introduced onto the silica nanoparticle surface by covalent bonds. A simple and efficient rotary evaporation method was adapted to load two different types of nitroimidazoles, pretomanid and MCC7433, with a high encapsulation efficiency of ≥86% and high drug loading of 8–10% w/w. The restricted nanopore environment allowed the compounds to remain in an amorphous state, providing enhanced solubility in most of the cases. The amino-functionalized MSNs improved the oral delivery of MCC7433, as determined by in vivo pharmacokinetic assessment in mice, making it a potential nanocarrier for other poorly soluble anti-TB drugs. This study also provides first insight into the use of amino functionalized MCM-41 to formulate pretomanid and a novel antitubercular compound, MCC7433 via a simple and scalable method. Given that most of the patients that suffered from tubercular and parasitic infectious diseases are from lower- and middle-income countries, MSN formulations that utilize simple preparation steps and are low cost can be highly desirable.

## Supporting Information Available

Synthesis of nitroimidazopyrazinone MCC7433, TEM images, thermograms of pure pretomanid and MCC7433, solubility study at 24 and 48 h (PDF)
