# Supplementary material for: Mesoporous Silica Nanoparticles Improve Oral Delivery of Antitubercular Bicyclic Nitroimidazoles
Source: ACS Biomater Sci Eng. 2021 Aug 31;8(10):4196–206. doi: 10.1021/acsbiomaterials.1c00807 (PMC9554870; doi:10.1021/acsbiomaterials.1c00807)
Supplement: Supplementary file 1 — ab1c00807_si_001.pdf [file ab1c00807_si_001.pdf]

Supporting information

Mesoporous silica nanoparticles improve oral delivery of antitubercular bicyclic nitroimidazoles

Chee Wei Ang[1,2], *Lendl Tan*[3,4], *Zhi Qu*[5,6], *Nicholas P. West*[3,4], *Matthew A. Cooper*[1,4], *Amirali Popat*[5,6*], *Mark A.T. Blaskovich*[1,4*]

[1] Centre for Superbug Solutions, Institute for Molecular Bioscience, The University of Queensland, St Lucia, Queensland 4072, Australia

[2] School of Science, Monash University Malaysia, 47500 Subang Jaya, Selangor, Malaysia

[3] School of Chemistry and Molecular Bioscience, The University of Queensland, St Lucia, Queensland 4072, Australia

[4] Australian Infectious Diseases Research Centre, St Lucia, Queensland 4067, Australia

[5] School of Pharmacy, The University of Queensland, Woolloongabba, Queensland 4102, Australia

[6] Mater Research Institute – The University of Queensland, Translational Research Institute, Woolloongabba, Queensland 4102, Australia

Table of Contents

Synthesis of nitroimidazopyrazinone, MCC7433 **S2**

Figure S1. TEM images of (a) MCC7433-MCM-41 and (b) pretomanid-MCM-41. **S2**

Figure S2. Reference thermograms of pure (a) pretomanid and (b) MCC7433. **S3**

Figure S3. Solubility of free and loaded pretomanid at 24 and 48 h (n = 3 ± SD). **S3**

References **S3**

Synthesis of nitroimidazopyrazinone, MCC7433

MCC7433, or 2-nitro-7-(4-(trifluoromethoxy)benzyl)imidazo[1,2-*a*]pyrazin-8(7*H*)-one, was synthesized via a five-step reaction as reported previously [1]. Characterization data are as follow: LCMS: R_t = 3.47 min, 99 A% @ 254 nm, $[M + H]^+$ = 355.0. ^{1}H NMR (600 MHz, DMSO-d_6) δ 8.82 (s, 1H), 7.62 (d, J = 5.9 Hz, 1H), 7.52 – 7.47 (m, 2H), 7.46 (d, J = 5.9 Hz, 1H), 7.39 – 7.33 (m, 2H), 5.16 (s, 2H).^{13}C NMR (150 MHz, DMSO-d_6) δ 152.8, 148.0, 147.7, 136.0, 135.1, 129.7, 123.5, 121.2, 120.0 (q, J = 257.6 Hz), 116.6, 107.5, 49.6. HRMS (ESI): *m/z* calcd for $C_{14}H_9F_3N_4NaO_4$ $[M + Na]^+$, 377.0468; found, 377.0467.

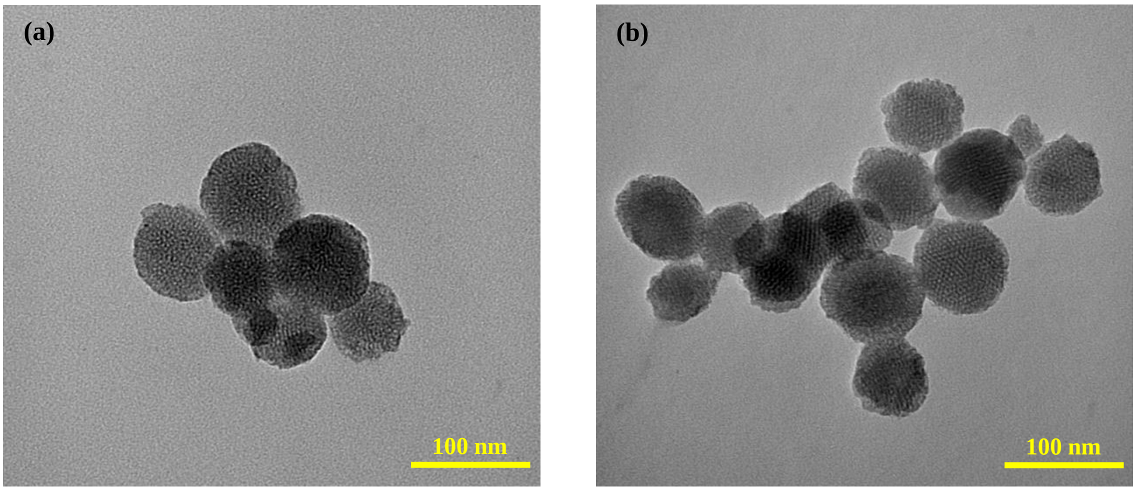

Figure S1. TEM images of (a) MCC7433-MCM-41 and (b) pretomanid-MCM-41.

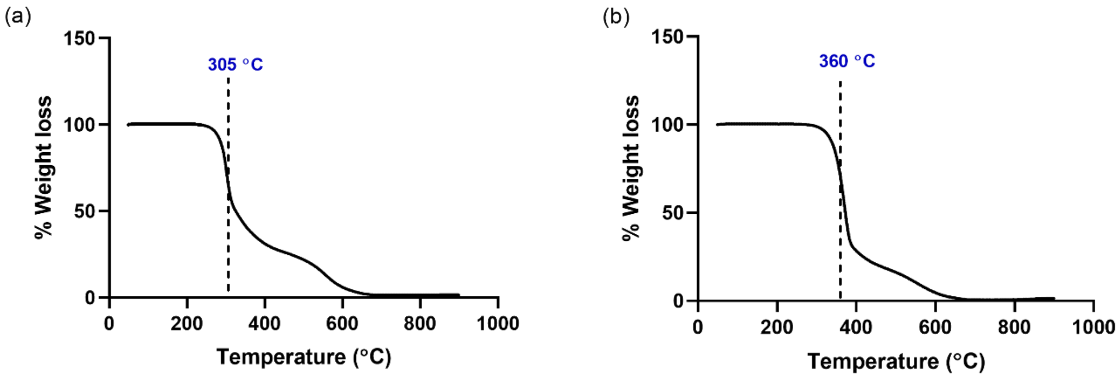

Figure S2. Reference thermograms of pure (a) pretomanid and (b) MCC7433.

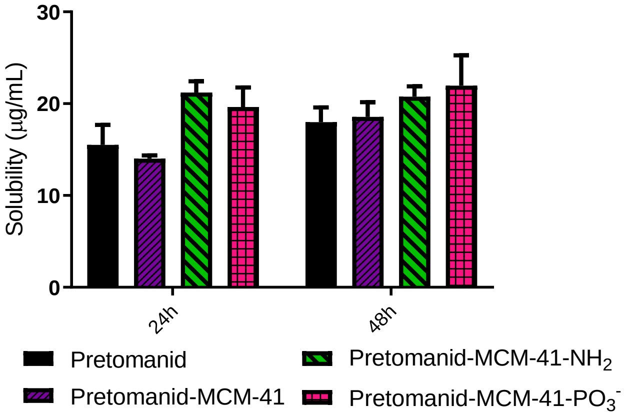

Figure S3. Solubility of free and loaded pretomanid at 24 and 48 h (n = 3 ± SD). No significant difference was observed for all samples at both time points, except for pretomanid-MCM-41 that showed 32% of improvement.

References

[1] A.M. Jarrad, C.W. Ang, A. Debnath, H.J. Hahn, K. Woods, L. Tan, M.L. Sykes, A.J. Jones, R. Pelingon, M.S. Butler, V.M. Avery, N.P. West, T. Karoli, M.A.T. Blaskovich, M.A. Cooper, Design, synthesis, and biological evaluation of 2-nitroimidazopyrazin-one/-es with antitubercular and antiparasitic activity, J. Med. Chem., 61 (2018) 11349-11371.
